# Hepatocellular proliferation in response to agonists of peroxisome proliferator-activated receptor *alpha: *a role for kupffer cells?

**DOI:** 10.1186/1477-3163-5-26

**Published:** 2006-11-27

**Authors:** Ibrahim A Alsarra, William G Brockmann, Michael L Cunningham, Mostafa Z Badr

**Affiliations:** 1 University of Missouri-Kansas City, Kansas City, MO 64108, USA; 2 Laboratory of Chemistry and Pharmacology, National Institute of Environmental Health Sciences, Research Triangle Park, NC 27709, USA

## Abstract

**Background:**

It has been proposed that PPARα agonists stimulate Kupffer cells in rodents which in turn, release mitogenic factors leading to hepatic hyperplasia, and eventually cancer. However, Kupffer cells do not express PPARα receptors, and PPARα agonists stimulate hepatocellular proliferation in both TNFα- and TNFα receptor-null mice, casting doubt on the involvement of Kupffer cells in the mitogenic response to PPARα agonists. This study was therefore designed to investigate whether the PPARα agonist PFOA and the Kupffer cell inhibitor methylpalmitate produce opposing effects on hepatocellular proliferation and Kupffer cell activity *in vivo*, in a manner that would implicate these cells in the mitogenic effects of PPARα agonists.

**Methods:**

Male Sprague-Dawley rats were treated intravenously *via *the tail vein with methylpalmitate 24 hrs prior to perfluorooctanoic acid (PFOA), and were sacrificed 24 hrs later, one hr after an intraperitoneal injection of bromodeoxyuridine (BrdU). Sera were analyzed for TNFα and IL-1β. Liver sections were stained immunohistochemically and quantified for BrdU incorporated into DNA.

**Results:**

Data show that PFOA remarkably stimulated hepatocellular proliferation in the absence of significant changes in the serum levels of either TNFα or IL-1β. In addition, methylpalmitate did not alter the levels of these mitogens in PFOA-treated animals, despite the fact that it significantly blocked the hepatocellular proliferative effect of PFOA. Correlation between hepatocellular proliferation and serum levels of TNFα or IL-1β was extremely poor.

**Conclusion:**

It is unlikely that mechanisms involving Kupffer cells play an eminent role in the hepatic hyperplasia, and consequently hepatocarcinogenicity attributed to PPARα agonists. This conclusion is based on the above mentioned published data and the current findings showing animals treated with PFOA alone or in combination with methylpalmitate to have similar levels of serum TNFα and IL-1β, which are reliable indicators of Kupffer cell activity, despite a remarkable difference in hepatocellular proliferation.

## Background

Treatment of rodents with agonists of PPARα results in liver cancer *via *mechanisms that remain unclear. While some studies implicate oxidative stress, caused by an overproduction of H_2_O_2_, as a consequence of peroxisome proliferation [1], others have suggested that these agonists increase rates of hepatocyte DNA synthesis leading eventually to the development of liver tumors [2,3].

In an attempt to delineate how PPARα agonists may initiate hepatocellular proliferation, investigators have proposed that these agonists stimulate Kupffer cells, the resident liver macrophages, which in turn release mitogenic factors leading to hepatic hyperplasia [2,4,5]. This conclusion is supported by the observation that presumed inactivation of Kupffer cells prevented the mitogenic effect of the PPARα agonist Wy-14,643 [4]. Furthermore, presence of nonparenchymal cells was required for replicative DNA synthesis in hepatocytes cultured in the presence of Wy-14,643 [6]. In addition, antibodies against tumor necrosis factor *alpha *(TNFα), presumably released by Kupffer cells upon their activation by agonists of PPARα, blocked the increase in liver cell replication in response to Wy-14,643. Finally, it was reported that TNFα suppressed apoptosis and induced DNA synthesis, effects which are similar to those produced by PPAR activators [7].

Results of a previous study in our laboratory do not support the aforementioned conclusions [8], and others [9] showed that activating the retinoid X receptors, the obligatory heterodimer of PPAR, inhibited TNFα production by isolated Kupffer cells. Importantly, it has been shown that Kupffer cells do not express PPARα receptors [10], and that PPARα agonists were able to stimulate hepatocellular proliferation in both TNFα- and TNFα receptor-null mice [11,12]. These findings cast doubt on the role of Kupffer cells in the hepatocellular proliferation known to occur in response to PPARα agonists in rodents.

Serum levels of TNFα and IL-1β are reliable indicators of the Kupffer cell activity status [13]. Thus, according to the hypothesis stipulating that PPARα agonists activate Kupffer cells as a prerequisite for the induction of hepatocellular proliferation, PFOA is expected to elevate serum levels of these mitogens. Also, it would be expected that hepatocellular proliferation and serum levels of TNFα or IL-1β will be blunted by methylpalmitate which is a known inhibitor of Kupffer cells. Data show that PFOA at a dose which remarkably stimulated hepatocellular proliferation did not produce significant changes in the serum levels of either TNFα or IL-1β. Furthermore, results reveal that Kupffer cell activity and PFOA-induced hepatocellular proliferation are unrelated phenomena, as evidenced by the fact that methylpalmitate did not diminish serum levels of TNFα or IL-1β when given in combination with PFOA, yet it significantly blocked the hepatocellular proliferative effect of this PPARα agonist. It is therefore unlikely that mechanisms involving the stimulation of Kupffer cells are responsible for the hyperplasia and the consequent hepatocarcinogenicity attributed to PPARα agonists in rodents.

## Methods

### Animal treatment and determination of serum TNFα, IL-1β and triglycerides

Male Sprague-Dawley rats (150–200 g) were purchased from Sasco (Omaha, NE). Animals received humane care in compliance with the National Research Council's criteria outlined in "Guide for the Care and Use of Laboratory Animals." Rats were treated intravenously *via *the tail vein with methylpalmitate (2 g/kg), or the 20% Tween 80+5% glucose vehicle, 48 hrs prior to sacrifice. Rats also received PFOA (100 mg/kg orally), or the corn oil vehicle, and were sacrificed 24 hrs later, one hr after an intraperitoneal injection of 100 mg/kg bromodeoxyuridine (BrdU) in 0.05 N NaOH. Animal sera were collected and analyzed for TNFα, IL-1β using commercially available kits (R&R Systems, Minneapolis, MN).

### Measurement of hepatic peroxisomal β-oxidation activity and cell proliferation

β-Oxidation assays were performed on liver homogenates as previously reported [14]. Briefly, liver samples were excised and homogenized in 0.25 M sucrose (20% w/v). Cyanide-insensitive palimtoyl-CoA oxidation was used as a measure of activity of peroxisomal β-oxidation enzymes, and was assayed by monitoring the rate of NAD^+ ^reduction spectrophotometrically at 240 nm using the method of Lazarow and DeDuve [15]. A unit of activity equals 1 μmol NAD^+ ^reduced/min.

A mid-lobe radial section of the right anterior lobe of the liver was flash frozen in liquid nitrogen, embedded in paraffin and serial tissue sections were mounted onto poly-1-lysine coated slides. Following deparaffination and dehydration, one set of slides was stained immunohistochemically for BrdU incorporation, routinely performed in our laboratories [16,17]. Random areas of the slides were chosen for counting stained and unstained hepatocytes (>1000 cells/animal).

### Statistical Analysis

Statistics were performed using one-way analysis of variance (ANOVA), or t-test, as appropriate P < .05 was considered significant.

## Results

### Effect of methylpalmitate on PFOA-induced hepatocellular proliferation and peroxisomal β-oxidation

**Figure 1:**
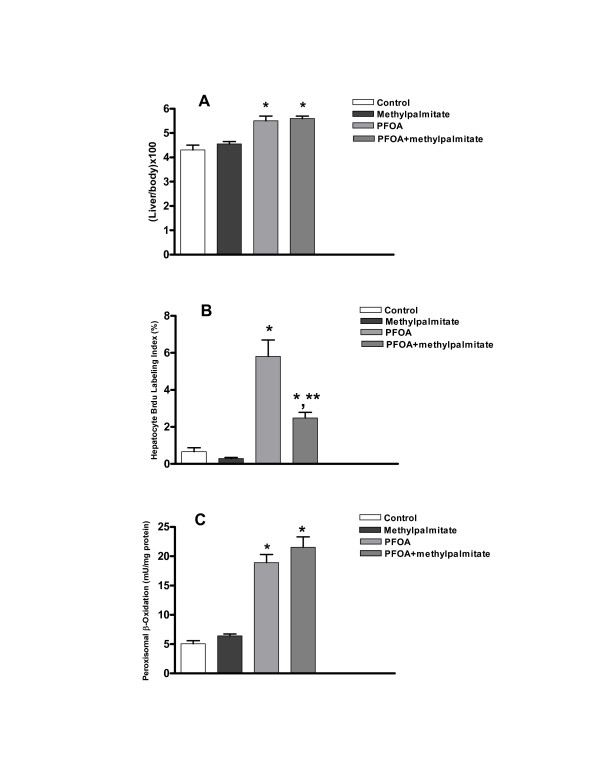
**Effect of methylpalmitate on liver/body weight ratios, hepatocellular proliferation and peroxisomal β-oxidation in response to the PPARα agonist PFOA**. Animals were treated, livers were excised, weighed, and liver/body weight ratios were calculated (**A**). Hepatocyte BrdU labeling indices (**B**), and peroxisomal β-oxiation activity (**C**) were determined as described under "Methods". Data are means ± SEM of 5 animals per group. *p < 0.05 compared to the control group. **p < .01 compared to the PFOA group.

PFOA significantly increased liver/body weight ratios from control values of 4.2 ± 0.2% to 5.5 ± 0.2%, an effect which was not altered by prior administration of methylpalmitate (Fig 1A). Since the increase in liver weight in response to PPAR agonists has a hyperplastic as well as a hypertrophic component, we investigated the effect of methylpalmitate on the hyperplastic component which is blamed for the hepatocarcinogenic effect of these chemicals. Methylpalmitate significantly reduced PFOA-induced levels of BrdU incorporation into hepatocye DNA (Fig 1B). Hepatocellular BrdU labeling index was 0.66 ± 0.21% in control rats (Fig 1B). PFOA increased labeling indices to 5.8 ± 0.9% (Fig 1B), and pretreatment with methylpalmitate diminished PFOA-induced labeling indices by 57% (Fig 1B).

In contrast to the significant inhibitory effect exerted by methylpalmitate on PFOA-induced hepatocellular proliferation, methylpalmitate did not influence the ability of this PPARα agonist to induce liver peroxisomal β-oxidation activity. PFOA caused a significant increase in peroxisomal β-oxidation activity from a basal value of 5.1 ± 0.53 mU/mg protein to19.1 ± 1.4 mU/mg protein (Fig 1C). Activity remained at 21.5 ± 1.8 mU/mg protein when methylpalmitate was given simultaneously with PFOA (Fig 1C).

### Serum TNFα and IL-1β Levels in Treated Rats

**Figure 2:**
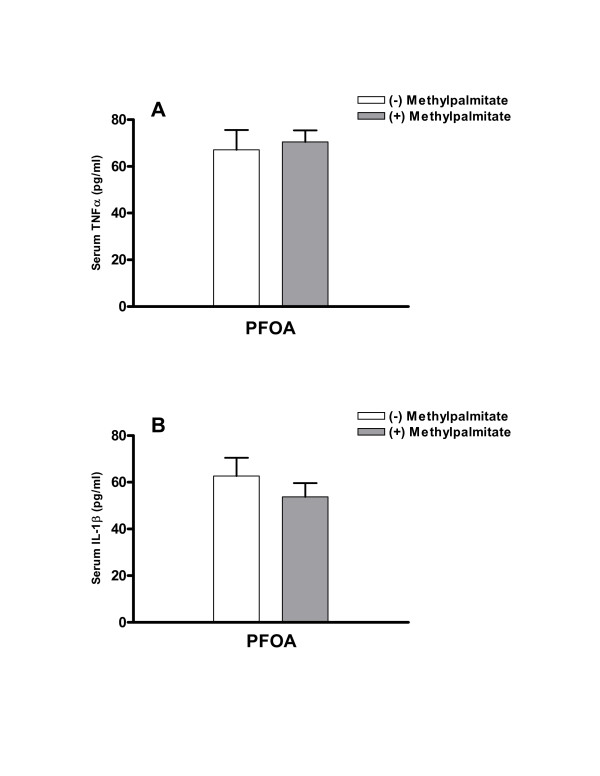
**Effect of methylpalmitate on serum levels of TNFα and IL-1β**. Animals were treated, and sera were collected and analyzed for TNFα (**A**) and IL-1β (**B**) as described under "Methods". Data are means ± SEM of 5 animals per group.

**Figure 3:**
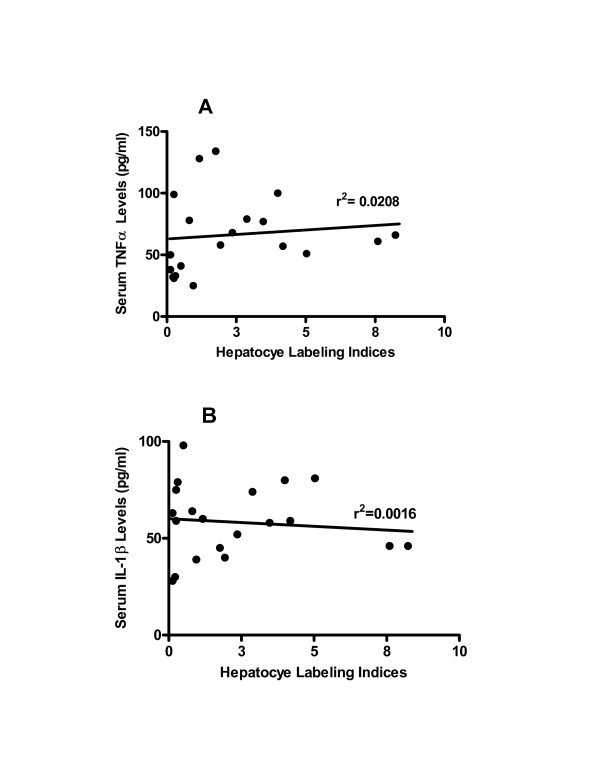
**Lack of correlation between BrdU labeling indices and serum mitogen levels**. Linear regression analyses were performed on data generated from animal groups depicted in Fig 1, for TNFα (**A**) and IL-1β (**B**).

In PFOA-treated animals, serum levels of TNFα were 67 ± 9 pg/ml (Fig 2). Treatment with methylpalmitate prior to PFOA did not exert detectable effects on these levels reaching 71 ± 5 pg/ml (Fig 2A). Similarly, serum levels of IL-1β in PFOA-treated rats of 62 ± 8 pg/ml were not significantly altered by prior administration of methylpalmitate, as levels were 54 ± 6 pg/ml (Fig 2B). In examining whether hepatocellular labeling indices correlated with serum levels of either TNFα or IL-1β, it was found that such a correlation did not exist, as r^2 ^values ranged from 0.021 to 0.0016, respectively (Fig 3).

## Discussion

Increased hepatocellular replication has been advanced as an important factor in liver cancer induced by PPARα agonists in rodents [18]. In an attempt to elucidate mechanisms involved in the hepatocellular proliferation caused by these agonists, studies have produced conflicting evidence with regards to the role of Kupffer cells in this process [19-23]. This study was therefore undertaken to test the hypothesis that hyperplasia induced by PPARα agonists correlates with Kupffer cell activity. The executed experiments examined the effect of the selective PPARα agonist, PFOA [24] on serum levels of TNFα and IL-1β, reliable indicators of Kupffer cell activity [13], in the absence and presence of methylpalmitate, known inhibitor of Kupffer cell activity [8].

Consistent with previous studies [25], PFOA caused significant hepatomegaly and hyperplasia in treated animals (Fig 1A &1B). This effect was observed in the absence of a concomitant effect on serum levels of TNFα and IL-1β (Fig 2). Furthermore, methylpalmitate which is a known inhibitor of Kupffer cell activity failed to modulate the effect of PFOA on TNFα and IL-1β serum levels (Fig 2), while significantly diminishing its hepatoproliferative effect (Fig 1B), dissociating these two phenomena. Indeed, examining the relationship between BrdU hepatocye labeling indices and serum TNFα and IL-1β levels in all treated animals revealed a very poor correlation (Fig 3), making the assertion that activation of Kupffer cells is a prerequisite for the hepatocellular proliferation caused by PPARα agonists a strenuous one.

It is noteworthy that results observed in this study with methylpalmitate mimic very closely those we obtained earlier with the mitochondrial inhibitor, rotenone [26]. In a previous study, we showed that rotenone inhibited hepatocellular proliferation in response to the PPARα agonist Wy-14,643 without interfering with the ability of this agonist to induce peroxisomal β-oxidation [26], an effect observed in this study for methylpalmitate with PFOA as the PPARα agonist (Fig 1B &1C). In addition, preliminary results (not shown) indicate that methylpalmitate produces effects on serum lipid metabolites and hepatocellular BrdU indices in the opposite direction from those caused by the natural congener, palmitic acid. This finding suggests that methylpalmitate may interfere with intermediary metabolism in a manner that diminishes the cellular ability to produce energy necessary for fueling cell proliferation. However, the exact mechanism by which this fatty acid derivative selectively inhibits hepatocyte, but not peroxisomal, proliferation in response to the PPARα agonists remains unclear, and requires further investigation.

## Authors' contributions

IA and WB treated animals, and collected tissues and sera, as well as performed peroxisomal β-oxidation assays. MLC measured serum mitogen levels, and performed BrdU labeling experiments. MB conceived, designed and coordinated the study.

## References

[B1] Reddy JK (1999). Carcinogenecity of peroxisome proliferators: evaluation and mechanisms. Biochem Soc Trans.

[B2] Marsman DS, Cattley RC, Conway JG, Popp JA (1988). Relationship of hepatic peroxisome proliferation and replicative DNA synthesis to the hepatocarcinogenicity of the peroxisome proliferators di(2-ethylhexyl) phthalate and [4-Chloro-6-(2,3-xylidino))-2-pyrimidinylthio]acetic acid (Wy-14,643) in rats. Cancer Res.

[B3] Soliman M, Cunningham M, Morrow J, Roberts LJ, Badr M (1997). Evidence against peroxisome proliferation-induced hepatic oxidative damage. Levels of esterified isoprostanes in livers of mice fed a diet containing [4-chloro-6-(2,3-xylidino)-2pyrimidinylthio]acetic acid (Wy-14,643). Biochem Pharmacol.

[B4] Rose M, Germolec DR, Schoonhoven R, Thurman RG (1997). Kupffer cells are casually responsible for mitogenic effect of peroxisome proliferators. Carcinogenesis.

[B5] Bojes HK, Germolec DR, Simeonova P, Bruccoleri A, Schoonhiven R, Luster MI, Thurman RG (1997). Antibodies to tumor necrosis factor α prevent increases in cell replication in liver due to the potent peroxisome proliferator, Wy-14,643. Carcinogenesis.

[B6] Karam WG, Ghanayem BI (1997). Induction of replicative DNA synthesis and PPAR alpha-dependent gene transcription by Wy-14 643 in primary rat hepatocyte and non-parenchymal cell co-cultures. Carcinogenesis.

[B7] Rolfe M, James N, Roberts R (1997). Tumor necrosis factor α (TNFα) suppresses apoptosis and induces DNA synthesis in rodent hepatocytes: a mediator of the hepatocarcinogenicity of peroxisome proliferators?. Carcinogenesis.

[B8] Youssef J, Badr M (1997). Activated Kupper cells attenuate the liver response to the peroxisome proliferator perfluorooctanoic acid. Mol Cell Biochem.

[B9] Uchimura K, Nakamuta M, Enjoji M, Irie T, Sugimoto R, Muta T, Iwamoto H, Hawata H (2001). Activation of retinoic X receptor and peroxisome proliferator-activated receptor-γ inhibits nitric oxide and tumor necrosis factor-α production in rat Kupffer cells. Hepatology.

[B10] Peters J, Rusyn I, Rose M, Gonzalez F, Thurman RG (2000). Peroxisome proliferator-activated receptor α is restricted to hepatic parenchymal cells, not kupffer cells: implications for the mechanism of action of peroxisome proliferators in hepatocarcinogenesis. Carcinogenesis.

[B11] Gilver B, Alberts D, Wollenberg G, Pitzenberger M, DeLuca J, Lawrence J (2000). TNFα is not required for Wy-14,643-induced proliferation in mice. Toxicologist.

[B12] Lawrence J, Wollenberg G, DeLuca J (2001). Tumor necrosis factor *alpha *is not required for Wy-14,643-induced cell proliferation. Carcinogenesis.

[B13] Rizzardini M, Zappone M, Villa P, Gnocchi P, Sironi M, Diomede L, Meazza C, Monshouwer M, Cantoni L (1998). Kupffer cell depletion partially prevents hepatic heme oxygenase 1 messenger RNA accumulation in systemic inflammation in mice: role of interleukin 1β. Hepatology.

[B14] Badr MZ (1992). Induction of peroxisomal enzyme activities by di-(2-ethylhexyl) phathalate in thyroidectomized rats with parathyroid transplants. J Pharmacol Exp Ther.

[B15] Lazarow PB, Deduve C (1997). A fatty acyl-CoA oxidizing system in rat liver peroxisomes: enhancement by clofibrate, a hypolipidemic drug. Proc Natl Acad Sci USA.

[B16] Cunningham ML, Fley J, Maronpot RR, Mattews HB (1991). Correlation of hepatocellular proliferation with hepatocarcinogenicity induced by the mutagenic noncarcinogen:carcinogen pair-2,6-and 2,4-diaminotoluene. Toxicol Appl Pharmacol.

[B17] Cunningham ML, Maronpot RR, Thompson M, Bucher JR (1994). Early responses of the liver of B6C3f1 mice to the hepatocarcinogen oxazepam. Toxicol Appl Pharmaco.

[B18] Kraupp-Grasl B, Huber W, Taper H, Schulte-Hermann R (1991). Increased susceptibility of aged rats to hepatocarcinogenesis by the peroxisome proliferator nafenopin and the possible involvement of altered liver foci occurring spontaneously. Cancer Res.

[B19] Klaunig J, Babich M, Baetcke K, Cook J, Corton C, David R, DeLuca J, Lai D, McKee R, Peters J, Roberts R, Fenner-Crisp P (2003). PPARα agonist-induced rodent tumors: modes of action and human relevance. Crit Rev Toxicol.

[B20] Holden P, Hasmall S, James N, West D, Brindle R, Gonzalez F, Peters J, Roberts R (2000). Tumor necrosis factor α (TNFα): role in suppression of apoptosis by the peroxisome proliferator nafenopin. Cell Mol Biol.

[B21] Rose M, Rusyn I, Bojes H, Germolec D, Luster M, Thurman RG (1999). Role of kupffer cells in peroxisome proliferator-induced hepatocyte proliferation. Drug Metab Rev.

[B22] Rusyn I, Tsukamoto H, Thurman RG (1998). Wy-14,643 rapidly activates nuclear factor κB in Kupffer cells. Carcinogenesis.

[B23] Rusyn I, Yamashina Y, Segal B, Schoonhoven R, Hollard S, Cattley R, Swenberg J, Thurman RG (2000). Oxidants from nicotinamide adenine dinucleotide phosphate oxidase are involved in triggering cell proliferation in the liver due to peroxisome proliferators. Cancer Res.

[B24] Maloney E, Waxman D (1999). trans-Activation of PPARα and PPARγ by structurally diverse environmental chamicals. Toxicol Appl Pharmacol.

[B25] Thottassery J, Winberg L, Youssef J, Cunningham ML, Badr M (1992). Regulation of perfluorooctanoic acid-induced peroxisomal enzyme activities and hepatocellular growth by adrenal hormones. Hepatology.

[B26] Cunningham ML, Soliman M, Badr M, Matthews H (1995). Rotenone, an anticarcinogen, inhibits cellular proliferation but not peroxisome proliferation in mouse liver. Cancer Lett.

